# The benefit of optimizing recanalization during mechanical thrombectomy in patients with acute ischemic stroke depends on preprocedural tissue-level collateralization

**DOI:** 10.1007/s00234-024-03443-7

**Published:** 2024-08-17

**Authors:** Paweł Wrona, Dominik Wróbel, Paweł Mizera, Joanna Jóźwik, Klaudia Jakobschy, Kaja Zdrojewska, Tomasz Homa, Katarzyna Sawczyńska, Tadeusz Popiela, Agnieszka Słowik, Wojciech Turaj

**Affiliations:** 1 Department of Neurology, Jagiellonian University Medical College, Krakow, Poland; 2 Department of Neurology, University Hospital, Krakow, Poland; 3 Faculty of Medicine, Student Scientific Group in Cerebrovascular Diseases, Jagiellonian University Medical College, Sw Anny 12 Street, Krakow, Poland; 4 Department of Radiology, University Hospital, Krakow, Poland; 5 Department of Radiology, Jagiellonian University Medical College, Krakow, Poland

**Keywords:** Mechanical thrombectomy, Tissue-level collateralization, Recanalization, Hypoperfusion intensity ratio, Ischemic stroke outcome

## Abstract

**Purpose:**

Thrombolysis in Cerebral Infarction (TICI) 3 represents the optimal angiographic outcome following mechanical thrombectomy (MT) for acute ischemic stroke (AIS). Although it is known to yield better outcomes than TICI 2b, the influence of preprocedural cerebral hemodynamics on the clinical advantage of TICI 3 over TICI 2b remains unexplored.

**Methods:**

This single-center retrospective analysis involved patients with anterior circulation AIS who underwent successful recanalization during MT at the Comprehensive Stroke Center, University Hospital, Krakow between January 2019 and July 2023. We assessed the benefit of achieving TICI 2c/3 over TICI 2b on the basis of preprocedural computed perfusion imaging results, primarily focusing on early infarct volume (EIV) and tissue-level collaterals indicated by hypoperfusion intensity ratio (HIR). Good functional outcome (GFO) was defined as a modified Rankin Score < 3 on day 90.

**Results:**

The study comprised 612 patients, of whom 467 (76.3%) achieved TICI 2c/3. GFO was more frequent in the TICI 2c/3 group (54.5% vs 69.4%, p < 0.001). There was interaction between the recanalization status and both HIR (Pi = 0.042) and EIV (Pi = 0.012) in predicting GFO, with disproportionately higher impact of HIR and EIV in TICI 2b group. The benefit from TICI 2c/3 over TICI 2b was insignificant among patients with good collaterals, defined by HIR < 0.3 (odds ratio:1.36 [0.58–3.18], p = 0.483).

**Conclusion:**

TICI 2c/3 improves patient functional outcomes compared to TICI 2b regardless of EIV. However, such angiographic improvement may be clinically futile in patients with good tissue-level collateralization. Our findings suggest that preprocedural HIR should be considered when optimization of recanalization is considered during MT.

## Introduction

Angiographic recanalization, defined as achieving a reperfusion score of 2b or higher on the Thrombolysis in Cerebral Infarction (TICI) scale, has long served as a critical prognostic indicator in patients undergoing mechanical thrombectomy (MT) for acute ischemic stroke (AIS). However, recent studies have demonstrated the limitations of this distinction, with near-complete (99%, TICI 2c) or complete (100%, TICI 3) recanalization associated with significantly improved functional outcomes compared with incomplete (TICI 2b) recanalization. This improvement translates to a net benefit of 15–20% in functional independence rates [1, 2]. Nevertheless, despite current ESO-ESMINT guidelines recommending the pursuit of TICI 3 angiographic scores during MT procedures [3], real-world data indicate that approximately 40% of procedures result in incomplete recanalization [4].

The functional benefits derived from optimizing recanalization from TICI 2b to TICI 2c/3 may be constrained by procedural prolongation and delayed reperfusion [5]. As more advanced procedural techniques emerge, such as intraarterial thrombolysis and microcatherers, enabling enhanced recanalization from TICI 2b to TICI 2c/3, neuroradiologists confront the clinical dilemma of whether to pursue complete recanalization despite potential trade-offs, such as increased thrombectomy device maneuvers and procedural time, or to terminate the procedure upon achieving incomplete angiographic results without prolonging it further [6]. While the quality of patient collateral circulation has been proposed as a modifying factor [5], it remains uncertain whether robust collateral circulation preserves the benefits of transitioning from TICI 2b to TICI 3 by maintaining cerebral tissue viability, or if it merely prevents tissue infarction, rendering such improvement futile.

Recent studies have established computed tomography perfusion-derived hypoperfusion intensity ratio (HIR) as an effective determinant of tissue-level collaterals, demonstrating good correlation with manual assessments of leptomeningeal collaterals and, in some instances, surpassing them [7–9]. Thus, we hypothesized that the degree of functional improvement resulting from higher recanalization scores depends on the quality of cerebral tissue-level collateralization. In addition, we sought to determine whether improvements in angiographic outcome lead to improved functional outcomes even in cohorts with significant baseline infarct core.

## Materials and methods

### Study population

We conducted a single-center, retrospective analysis of data collected prospectively from patients with AIS admitted to the Comprehensive Stroke Center, University Hospital, Krakow from January 2019 to July 2023. We included patients who met the following criteria: (1) age ≥ 18 years; (2) diagnosis of AIS consistent with the AHA/ASA definition [10]; (3) large vessel occlusion of the internal carotid artery, M1 or M2 segment of the middle cerebral artery, or tandem occlusion diagnosed with the use of computed tomography angiography or magnetic resonance angiography; (4) MT performed within 6 h from symptom onset, or between 6 and 24 h from time last known well according to ESO-ESMINT guidelines on MT in AIS [3]; (5) achieved successful recanalization during MT, denoted by TICI score 2b or higher. We excluded patients in whom perfusion imaging was not performed or non-diagnostic (mainly due to motion artifacts) or patients who lacked 90-day follow-up. We additionally excluded patients presenting < 90 min from last known well, because they can present significant overestimation of early infarct volume [11].

All patients were treated according to the selection and eligibility criteria of the current European Stroke Organization (ESO) guidelines on intravenous thrombolysis and mechanical thrombectomy in acute ischemic stroke [12]. All procedures were performed in accordance with the Declaration of Helsinki [13].

### Data collection

The following data were collected from all patients: age and sex, time last known well before stroke, chronic antiplatelet or anticoagulant use, risk factors for stroke: hypertension, diabetes mellitus, atrial fibrillation, smoking status (smokers were those who were either smoking while they were recruited into the study or smoked in the past), history of stroke or transient ischemic attack, and hyperlipidemia. The National Institutes of Health Stroke Scale (NIHSS) scale on admission was used to assess stroke severity. Stroke etiology was classified according to the Trial of ORG 10,172 in Acute Stroke Treatment (TOAST) criteria [14]. Medical complications that occurred after mechanical thrombectomy were diagnosed as described elsewhere [15].

Patients with AIS qualified for MT underwent the following protocol of imaging studies: noncontrast computed tomography (Alberta Stroke Programme Early CT Score—ASPECT), computed tomography angiography, and computed tomography perfusion with post-processing analysis using RAPID (iSchemaView, Menlo Park, CA) software. The following perfusion parameters were recorded: volume of cerebral blood flow (CBF) less than 30% (early infarct volume), time to peak concentration-more-than-6-s volume (T6_max_), T10_max_, and volume of T6_max_ minus CBF < 30% (penumbra). Tissue-level collaterals quality was determined by hypoperfusion intensity ratio (defined as ratio of T10max/T6max), with higher HIR denoting more severe delay in the contrast bolus arrival time and thus signifying worsening collaterals quality. Good tissue-level collateralization was defined as HIR < 0.3 [16, 17].

For each patient, we calculated the time from last known well to imaging and groin puncture. MT was performed either via thromboaspiration, stent-retriever or combined approach. Follow-up noncontrast computed tomography was performed 24 h after mechanical thrombectomy (Siemens Somatom Definition Edge 64-detector scanner, Siemens Healthineers, Forchheim, Germany).

Immediate recanalization was graded according to the extended TICI (eTICI) criteria: grade 0 —no perfusion noted (0% reperfusion); grade 1 — reduction in thrombus but without any resultant filling of distal arterial branches; grade 2a — reperfusion of 1–49% of the territory; 2b — partial perfusion, 50–89%; grade 2c — extensive reperfusion of 90–99% of the territory; grade 3 — complete or full reperfusion (100% reperfusion).

The following parameters related to mechanical thrombectomy were recorded: site of the occlusion, presence of any intracerebral hemorrhage on noncontrast computed tomography performed 24 h after mechanical thrombectomy, presence of symptomatic intracerebral hemorrhage (sICH) defined according to ECASS 2 criteria [18], and successful recanalization after mechanical thrombectomy defined as an eTICI score of 2b or 3.

Disability 90 days after the onset of ischemic stroke was assessed using the modified Rankin Scale (mRS) during scheduled visits in an outpatient clinic or through telephone interviews with patients or their family members/caregivers. A good functional outcome was defined as mRS score < 3 on day 90.

### Statistical analysis

Categorical variables were presented as counts and percentages, while continuous variables were described using the median and interquartile range (IQR) because of the lack of normal distribution, as indicated by the Kolmogorov–Smirnov test. The significance of differences between groups was assessed using the χ^2^ test for categorical variables and the Mann–Whitney *U*-test for continuous variables. Initially, univariate analysis was conducted to explore differences in baseline characteristics between patients with TICI 2b and TICI 2c/3 recanalization scores. Subsequently, multivariate analysis was performed to evaluate the predictive performance of the CTP-derived parameters for the 90-day good functional outcome. Additionally, to assess potential interactions between recanalization status and perfusion deficits, an interaction term (recanalization score*perfusion parameter) was introduced into each model, and the corresponding P-value for interaction was reported. These models were adjusted for important confounders, including patient age, history of previous stroke/transient ischemic attack, baseline NIHSS score, thrombolytic treatment, stroke etiology, serum glucose level, first pass effect, presence of hemorrhagic transformation, and occurrence of stroke-related pneumonia and urinary tract infection.

In the final stage, we stratified our population into two subgroups based on the presence of good collateral status (HIR < 0.3), and multivariate analyses were conducted once more to determine the significance of recanalization status in predicting a good functional outcome at 90 days. These models were adjusted for the confounding variables previously mentioned.

For each variable we report the odds ratio (OR) and 95% confidence intervals (CI). A two-sided p-value of less than 0.05 was considered significant. All analyses were performed using IBM SPSS 9 (Chicago, IL) and STATA SE 18 (StataCorp LLC, TX).

## Results

**Fig. 1:**
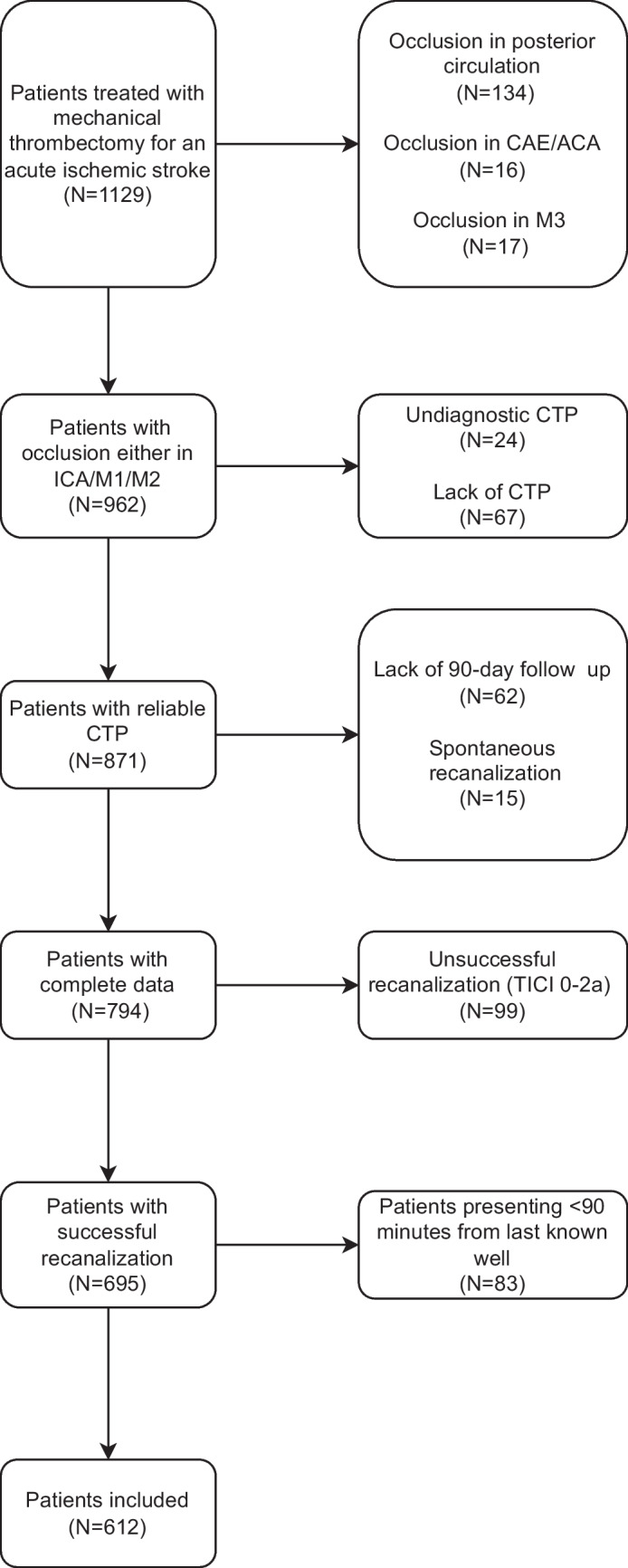
Study flowchart. Abbreviations: ACA – anterior cerebral artery; CAE – external carotid artery; CTP – computed tomography perfusion; ICA – internal carotid artery; M1, M2, M3 – segments of middle cerebral artery

In total, 612 patients were enrolled, with 467 (76.31%) attaining a TICI score of 2c/3. The study flowchart is presented in Fig. 1. Generally, patients achieving a final score of TICI 2b did not differ significantly in baseline characteristics and complications compared to those achieving TICI 2c/3. Regarding the procedure, patients with TICI 2b had more frequent combined (thromboaspiration + stent-retriever) approach; however, they experienced a less frequent occurrence of the first pass effect during MT and a good functional outcome at 90 days. Detailed characteristics are outlined in Table 1.

**Table 1:** Comparison of baseline characteristics between patients with acute ischemic stroke who achieved recanalization graded as TICI 2b or TICI 2c/3

	Patients with TICI 2b (N = 145)	Patients with TICI 2c/3 (N = 467)	*P-*value
Demographics			
Age [years], median (IQR)	70 (60–80)	73 (65–81)	0.171
Stroke risk factors			
Hypertension, n (%)	97 (66.6)	345 (73.9)	0.101
Diabetes mellitus, n (%)	35 (24.1)	107 (22.9)	0.76
Hyperlipidemia, n (%)	33 (22.8)	93 (19.9)	0.459
Atrial fibrillation, n (%)	38 (26.2)	159 (34)	0.078
History of stroke/TIA, n (%)	16 (11)	64 (13.7)	0.405
History of myocardial infarction, n (%)	5 (20)	48 (32.9)	0.198
Smoking, n (%)	20 (13.8)	105 (22.5)	0.075
Clinical characteristics			
Baseline NIHSS score, median (IQR)	16 (12–19)	16 (10–19)	0.311
Time from last known well to puncture [minutes], median (IQR)	310 (255–390)	306 (240–383)	0.482
Intravenous thrombolysis, n (%)	80 (55.2)	248 (53.1)	0.663
Serum glucose concentration [mmol/L], median (IQR)	6.3 (5.4–7.8)	6.2 (5.1–7.7)	0.218
Procedural characteristics			
First pass effect, n (%)	66 (45.5)	268 (57.4)	0.012
Type of device used			< 0.001
Stent retiever, n (%)	14 (9.7)	15 (3.2)	
Thromboaspiration, n (%)	54 (37.2)	250 (53.5)	
Combined, n (%)	77 (53.1)	202 (43.3)	
Occluded artery			0.183
ICA, n (%)	21 (14.5)	66 (14.1)	
M1, n (%)	78 (53.8)	281 (60.2)	
M2, n (%)	29 (20)	90 (19.3)	
Tandem occlusion, n (%)	17 (11.7)	30 (6.4)	
Stroke etiology			0.279
Cardioembolic, n (%)	54 (37.2)	215 (46)	
Large vessel disease, n (%)	26 (17.9)	74 (15.8)	
Undetermined, n (%)	58 (40)	163 (34.9)	
Rare, n (%)	7 (4.8)	15 (3.2)	
Neuroimaging-related variables			
Baseline ASPECTs, median (IQR)	8 (6–9]	8 (7–9)	0.069
Penumbra volume [mL], median (IQR)	39 (55–122)	36 (53–126)	0.287
Infarct volume (CBF < 30%) [mL], median (IQR)	9 (0–35)	8 (0–26)	0.667
HIR, median (IQR)	0.36 (0.19–0.52)	0.33 (0.17–0.5)	0.323
Stroke-related complications			
Pneumonia, n (%)	35 (24.1)	92 (19.7)	0.25
Urinary tract infection, n (%)	29 (20)	68 (14.6)	0.117
Recurrent stroke, n (%)	21 (52.5)	60 (41.7)	0.222
Hemorrhagic transformation, n (%)	43 (29.7)	110 (23.6)	0.138
Symptomatic hemorrhagic transformation, n (%)	12 (8.3)	27 (5.8)	0.283
Outcomes			
90-day good functional outcome (mRS < 3), n (%)	79 (54.5)	324 (69.4)	< 0.001

Abbreviations: ASPECTs—Alberta Stroke Programme Early CT score; CBF – cerebral blood flow; HIR – hypoperfusion intensity ratio; ICA – internal carotid artery; IQR – interquartile range; M1, M2 – segments of the middle cerebral artery; mRS – modified Rankin scale; NIHSS – National Institutes of Health Stroke Scale; TIA – transient ischemic attack; TICI – Thrombolysis in Cerebral Infarction

### Impact of CTP parameters on the 90-day outcome according to angiographic recanalization status

**Fig. 2:**
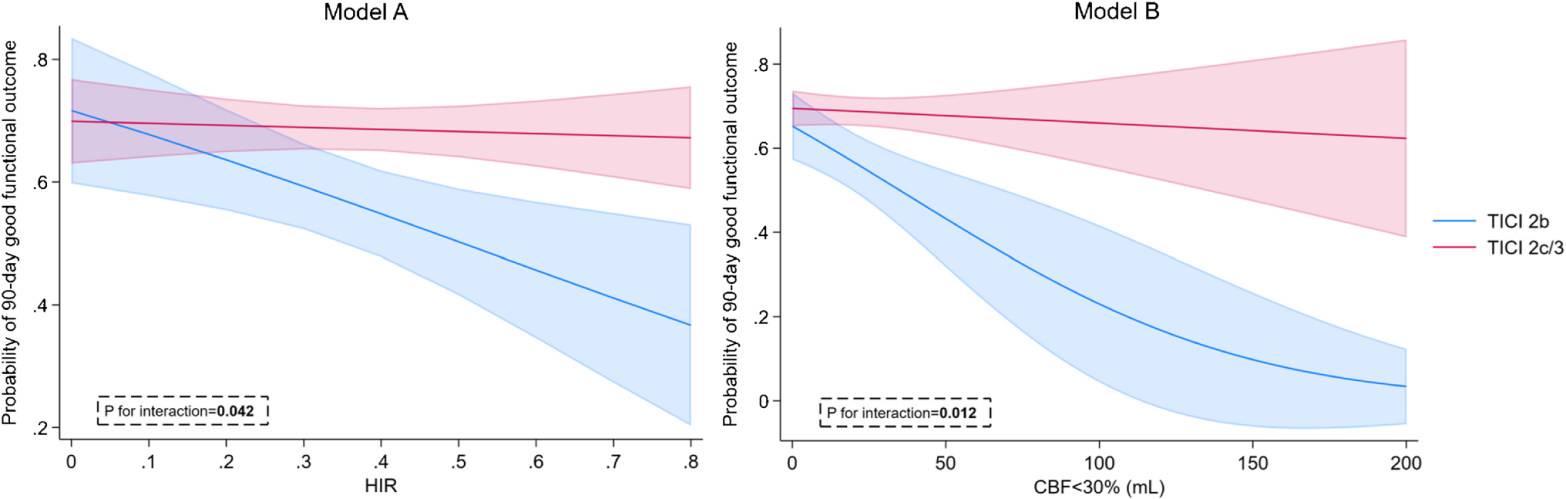
Impact of early infarct volume on 90-day good functional outcome according to recanalization status (logistic regression models). HIR – hypoperfusion intensity ratio; CBF – cerebral blood flow. Model A: Association between HIR and good outcome according to patient recanalization score. Model B: Association of early infarct volume (CBF < 30%) and good outcome according to patient recanalization score. Models are adjusted for age, previous stroke/transient ischemic attack, baseline National Institutes of Health Stroke Scale score, intravenous thrombolysis, serum glucose level, first pass effect, hemorrhagic transformation, stroke-related pneumonia, urinary tract infection, and stroke etiology

Multivariate analysis revealed the independent predictive significance of both total hypoperfusion and early infarct volume on the achievement of a good functional outcome at 90 days. In addition, a significant interaction was observed between binary recanalization status (TICI 2b vs TICI 2c/3) and both HIR (P for interaction = 0.042) and early infarct volume (P for interaction = 0.012) (Table 2). In the TICI 2b group, each 0.1 increment in HIR resulted in a 0.76 decrease in the odds of achieving a good outcome (95% CI: 0.61–0.94, p = 0.011), whereas in the TICI 2c/3 group, such a decrease was not significant (OR: 0.97, 95% CI: 0.87–1.1, p = 0.670). Similarly, for each 10 ml increase in early infarct volume, the odds of achieving a good outcome decreased disproportionately in the TICI 2b group (OR: 0.76, 95% CI: 0.64–0.91, p = 0.002) compared with the TICI 2c/3 group (OR: 0.97, 95% CI: 0.9–1.06, p = 0.551). The enhanced rates of good functional outcome associated with a TICI 2c/3 recanalization score were evident only after a transition to a higher HIR. This relationship was not observed in the case of early infarct volume, where the benefit of TICI 2c/3 was consistent across all ranges of infarct volume (Fig. 2).

**Table 2:** The impact of perfusion parameters on the odds ratio of a 90-day good functional outcome and their interaction with recanalization score

	Odds ratio (adjusted 95% confidence interval)	Adjusted* p-value	*P*-value for interaction
Hypoperfusion intensity ratio (per 0.1)	0.92 (0.83–1.02)	0.097	**0.042**
Penumbra volume (per 10 mL)	0.98 (0.94–1.01)	0.188	0.974
Early infarct volume (per 10 mL)	0.92 (0.86–0.997)	**0.038**	**0.012**
Hypoperfused tissue (per 10 mL)	0.97 (0.94–0.997)	**0.033**	0.234

^*^Models are adjusted for age, previous stroke/transient ischemic attack, baseline National Institutes of Health Stroke Scale score, intravenous thrombolysis, serum glucose level, first pass effect, hemorrhagic transformation, stroke-related pneumonia, urinary tract infection, and stroke etiology

### Subanalysis of patients benefiting from TICI 2c/3 in subgroups dichotomized according to HIR

**Fig. 3:**
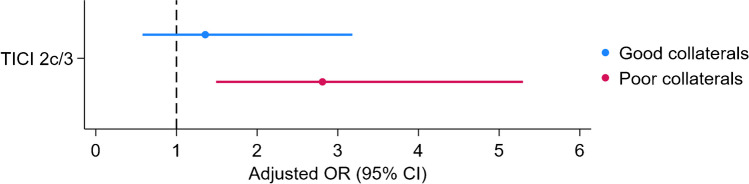
Comparison of benefit from TICI2c/3 over TICI 2b between patients with good (hypoperfusion intensity ratio [HIR] < 0.3) versus poor (HIR ≥ 0.3) collateral circulation. Models are adjusted for age, previous stroke/transient ischemic attack, baseline National Institutes of Health Stroke Scale score, intravenous thrombolysis, serum glucose level, first pass effect, hemorrhagic transformation, stroke-related pneumonia, urinary tract infection, and stroke etiology

When tissue-level collateralization was categorized as good (HIR < 0.3) and poor (HIR ≥ 0.3), adjusted analysis demonstrated that there is no benefit of TICI 2c/3 over TICI 2b in patients with good collaterals (OR: 1.357, 95% CI: 0.58–3.18, p = 0.483). However, a significant benefit was observed in the group with poor collaterals (OR: 2.8, 95% CI: 1.49–5.29, p = 0.001) (Fig. 3).

## Discussion

Analysis of our substantial single-center cohort of patients who underwent CTP imaging revealed three key findings. First, patients with successful yet incomplete angiographic recanalization experience a disproportionately negative impact from higher HIR, indicating deteriorating tissue-level collateral quality, and from increasing early infarct volume compared with those completely recanalized. Second, the benefit from complete recanalization diminishes to insignificance in patients with good collateral circulation. Finally, the benefit derived from complete recanalization becomes even more pronounced with an increase in the early infarct volume.

As anticipated, in patients with a reperfusion score of TICI 2b, lower HIR was independently associated with significantly improved functional outcomes at 90 days, although such an effect was not observed in the TICI 2c/3 group. Our findings underscore the intricate relationship between cerebral tissue-level collaterals and hemodynamics with the final angiographic outcome. In cases where collaterals are compromised, the prognosis of patients with incomplete recanalization deteriorates markedly, as the restoration of blood flow is incomplete, leading to eventual infarction of tissue reliant on insufficient supply from poorly developed collaterals. Conversely, well-functioning collaterals can sustain ischemic tissue, thereby diminishing the benefits of TICI 3 reperfusion [5]. This observed disparity offers new insights into the interpretation of tissue-level collaterals indicated by HIR in the context of the extent of patient revascularization and should be considered in future trials focusing on cerebral collateralization in endovascularly treated patients.

The findings presented align with those of a previous study that demonstrated the relevance of HIR in predicting both functional outcome and the formation of malignant cerebral edema in patients who did not achieve successful recanalization, specifically those with TICI ranging from 0 to 2a [19]. Our results can be viewed as an extension of this prior study, focusing specifically on patients in whom the procedure was deemed "successful". Furthermore, we offer clinically pertinent insights indicating that a large proportion of patients with TICI 2b still achieve a good functional outcome, particularly if they exhibit good collateral circulation. Importantly, when patients were stratified into subgroups based on dichotomization set at an HIR level of 0.3, it was evident that in the subgroup with good collateralization, the benefit from TICI 2c/3 over TICI 2b did not reach significance, in contrast to the group with poor collateralization. A similar relationship was recently reported, although the authors used manual assessment of collaterals, which involves time-consuming evaluation and inherently introduces inter-rater variability [20].

The findings described here have direct implications for clinical practice. When a patient exhibits particularly robust collaterals, as indicated by an HIR < 0.3, achieving a final recanalization score of TICI 2b is non-inferior to achieving TICI 3 in terms of functional outcome. Therefore, in such cases, our evidence supports the consideration of concluding the procedure without justification for a higher number of passes of MT devices or prolongation of the procedure, both of which are known to have adverse effects on functional outcomes and increase the risk of hemorrhage [21, 22]. Additionally, the benefit derived from TICI 3 recanalization diminishes with the number of MT maneuvers, and it has been demonstrated that outcomes after the third retrieval attempt are comparable to those of first-pass TICI 2b [6]. In conjunction with our findings, this suggests that performing additional thrombectomy maneuvers may be clinically futile, particularly in patients with optimal tissue microperfusion.

Notably, in our study, tissue-level collateralization was not directly associated with the final angiographic result itself, contrary to a previous report indicating a higher likelihood of complete recanalization in patients with good arterial collaterals [23]. This difference may stem from the use of a different collateral marker in our study, which focused more on tissue-level hemodynamics than visible leptomeningeal collateralization. Furthermore, this discrepancy may also be attributed in part to the broader inclusion criteria in our study, as we included patients presenting with occlusion localized in the M2 segment of the middle cerebral artery. However, the impact of collateral hemodynamics on the achievement of complete recanalization warrants further exploration.

The observation of a disproportionately higher impact of early infarct volume on the functional status of patients with a TICI 2b recanalization score can be interpreted from two perspectives. First, it underscores the prognostic significance of early infarct volume in such patients, with the benefit from MT decreasing markedly as infarct size increases—a trend that is considerably less relevant for patients with a TICI 2c/3 score. However, it is important to acknowledge the challenges associated with interpreting early infarct volume, often referred to as "ghost infarct core" which tends to occur in patients imaged shortly after symptom onset [24]. To address this issue, we chose to include only patients presenting more than 90 min from their last known well time, as this subgroup has been shown not to present significant ghost infarct core phenomenon [11].

Conversely, the reported relationship offers illuminating insights into the ongoing debate regarding whether high recanalization scores confer greater benefits in patients with large infarct cores upon admission. Indeed, our findings suggest that the benefit may be at least noninferior with increasing early infarct volume. However, this observed effect contrasts with the results of a previous study where the authors reported no significant functional benefit between TICI 3 and TICI 2b in patients with large infarct cores. It is important to note that in that study, a large core was defined using the ASPECTS score of 3–5 [25], which may not provide as precise information on cerebral tissue viability as perfusion imaging. Importantly, only 80 patients in our study cohort presented with early infarct volumes large enough to be considered substantial (≥ 50 mL). Thus, statistical robustness of that finding cannot be considered sufficient. Consequently, further studies are warranted to conclusively determine whether such benefits indeed exist.

Additionally, our results revealed lower frequency of the first pass effect with concomitant more frequent combined approach to the MT procedure in TICI 2b group. Such results were expected, as the poorer recanalization outcome is usually associated with higher recanalization attempts [23]. Moreover, in our Center first-line approach to the thrombectomy in the great majority of cases is thromboaspiration; stent-retriever attempts are performed afterwards the aspiration maneuver fails. Thus, as the first-pass effect was observed predominantly in the TICI 2c/3 group, so was the sole thromboaspiration approach. Despite differences in intra-procedural characteristics, the groups did not differ in case of any and symptomatic intracerebral hemorrhage; the type of device used was not linked with sICH either.

Our study has several notable limitations. First, it is retrospective in nature, which may introduce inherent biases. Second, the limited number of patients with sufficient early infarct volume to be classified as "large" restricted our ability to conduct robust analyses within this subgroup. Additionally, the exact number of passes during the MT procedure was not available for all patients, preventing us from exploring its potential interplay with angiographic scores and perfusion parameters. Lastly, pre-stroke (mRS) scores were not systematically collected before 2021 and therefore could not be incorporated into our multivariate models. However, patients treated with MT at our facility typically have pre-stroke mRS scores of 0–1. The observational nature of the current study highlights the need for future randomized trials to definitively assess the impact of perfusion imaging results on the decision-making process during MT.

In summary, our study underscores the importance of considering computed tomography perfusion results, particularly focusing on tissue-level collateralization indicated by HIR, especially in decision about further optimizing the reperfusion score from TICI 2b via methods like intraarterial thrombolysis. As demonstrated, patients with good collateralization do not derive functional benefits from a TICI 2c/3 score over TICI 2b. Therefore, pursuing such a result in these patients may not be necessary.

## Data Availability

Data that confirms our findings is available from corresponding author upon reasonable request.

## References

[1] Rizvi A, Seyedsaadat SM, Murad MH, Brinjikji W, Fitzgerald ST, Kadirvel R et al (2019) Redefining “success”: a systematic review and meta-analysis comparing outcomes between incomplete and complete revascularization. J Neurointerv Surg 11(1):9–13. 10.1136/neurintsurg-2018-013950 29802163 10.1136/neurintsurg-2018-013950

[2] Kaesmacher J, Dobrocky T, Heldner MR, Bellwald S, Mosimann PJ, Mordasini P et al (2018) Systematic review and meta-analysis on outcome differences among patients with TICI2b versus TICI3 reperfusions: success revisited. J Neurol Neurosurg Psychiatry 89(9):910–917. 10.1136/jnnp-2017-317602 29519899 10.1136/jnnp-2017-317602 PMC6109240

[3] Turc G, Bhogal P, Fischer U, Khatri P, Lobotesis K, Mazighi M et al (2019) European Stroke Organisation (ESO) - European Society for Minimally Invasive Neurological Therapy (ESMINT) Guidelines on Mechanical Thrombectomy in Acute Ischaemic StrokeEndorsed by Stroke Alliance for Europe (SAFE). Eur stroke J 4(1):6–12. 10.1177/2396987319832140 31165090 10.1177/2396987319832140 PMC6533858

[4] Hill MD, Goyal M, Menon BK, Nogueira RG, McTaggart RA, Demchuk AM et al (2020) Efficacy and safety of nerinetide for the treatment of acute ischaemic stroke (ESCAPE-NA1): a multicentre, double-blind, randomised controlled trial. Lancet 395(10227):878–887. 10.1016/S0140-6736(20)30258-0 32087818 10.1016/S0140-6736(20)30258-0

[5] Kaesmacher J, Ospel JM, Meinel TR, Boulouis G, Goyal M, Campbell BCV et al (2020) Thrombolysis in Cerebral Infarction 2b Reperfusions: To Treat or to Stop? Stroke 51(11):3461–3471. 10.1161/STROKEAHA.120.030157 32993461 10.1161/STROKEAHA.120.030157

[6] Flottmann F, van Horn N, Maros ME, McDonough R, Deb-Chatterji M, Alegiani A et al (2022) Early TICI 2b or Late TICI 3—Is Perfect the Enemy of Good? Clin Neuroradiol 32(2):353. 10.1007/s00062-021-01048-8 34191040 10.1007/s00062-021-01048-8 PMC9187567

[7] Guenego A, Fahed R, Albers GW, Kuraitis G, Sussman ES, Martin BW et al (2020) Hypoperfusion intensity ratio correlates with angiographic collaterals in acute ischaemic stroke with M1 occlusion. Eur J Neurol 27(5):864–870. 10.1111/ene.14181 32068938 10.1111/ene.14181

[8] Wan Z, Meng Z, Xie S, Fang J, Li L, Chen Z et al. (2022) Correlation between Hypoperfusion Intensity Ratio and Functional Outcome in Large-Vessel Occlusion Acute Ischemic Stroke: Comparison with Multi-Phase CT Angiography. J Clin Med 11(18). 10.3390/jcm12123990 10.3390/jcm11185274 PMC9503156 36142924

[9] Lyndon D, van den Broek M, Niu B, Yip S, Rohr A, Settecase F (2021) Hypoperfusion Intensity Ratio Correlates with CTA Collateral Status in Large-Vessel Occlusion Acute Ischemic Stroke. AJNR Am J Neuroradiol 42(8):1380–1386. 10.3174/ajnr.A7181 34140276 10.3174/ajnr.A7181 PMC8367626

[10] Sacco RL, Kasner SE, Broderick JP, Caplan LR, Connors JJ, Culebras A et al (2013) An Updated Definition of Stroke for the 21st Century. Stroke 44(7):2064–2089. 10.1161/STR.0b013e318296aeca 23652265 10.1161/STR.0b013e318296aeca PMC11078537

[11] Sarraj A, Campbell BCV, Christensen S, Sitton CW, Khanpara S, Riascos RF et al (2022) Accuracy of CT Perfusion-Based Core Estimation of Follow-up Infarction: Effects of Time Since Last Known Well. Neurology 98(21):E2084–E2096. 10.1212/WNL.0000000000200269 35450966 10.1212/WNL.0000000000200269 PMC9169942

[12] Berge E, Whiteley W, Audebert H, Marchis GM De, Fonseca AC, Padiglioni C, et al. (2021) European Stroke Organisation (ESO) guidelines on intravenous thrombolysis for acute ischaemic stroke. Eur Stroke J 6(1):I. 10.1177/2396987321989865 10.1177/2396987321989865 PMC7995316 33817340

[13] World Medical Association Declaration of Helsinki: ethical principles for medical research involving human subjects. (2013) JAMA 310(20):2191–4. 10.1001/jama.2013.281053 10.1001/jama.2013.281053 24141714

[14] HP A, BH B, LJ K, J B, BB L, DL G, et al. (1993) Classification of subtype of acute ischemic stroke. Definitions for use in a multicenter clinical trial. TOAST. Trial of Org 10172 in Acute Stroke Treatment. Stroke 24(1):35–41. 10.1161/01.STR.24.1.35 10.1161/01.str.24.1.35 7678184

[15] Nowak K, Derbisz J, Pęksa J, Łasocha B, Brzegowy P et al (2020) Post-stroke infection in acute ischemic stroke patients treated with mechanical thrombectomy does not affect long-term outcome. Adv Interv Cardiol 4(62):452–459. 10.5114/aic.2020.101771 10.5114/aic.2020.101771 PMC7863840 33598019

[16] Guenego A, Farouki Y, Mine B, Bonnet T, Hulscher F, Wang M et al (2022) Hypoperfusion Intensity Ratio Predicts Infarct Growth After Successful Thrombectomy for Distal Medium Vessel Occlusion. Clin Neuroradiol 32(3):849–856. 10.1007/s00062-022-01141-6 35166857 10.1007/s00062-022-01141-6

[17] Zhang Q, Yang S, Cheng XD, Sun H, Li BH, Yu NW (2023) Cerebral blood volume index can predict the long-term prognosis after endovascular thrombectomy in patients with acute ischemic stroke due to large vessel occlusion. J Clin Neurosci 117:120–124. 10.1016/j.jocn.2023.09.030 37801876 10.1016/j.jocn.2023.09.030

[18] Hacke W, Kaste M, Fieschi C, Von Kummer R, Davalos A, Meier D et al (1998) Randomised double-blind placebo-controlled trial of thrombolytic therapy with intravenous alteplase in acute ischaemic stroke (ECASS II). Lancet 352(9136):1245–1251. 10.1016/S0140-6736(98)08020-9 9788453 10.1016/s0140-6736(98)08020-9

[19] Murray NM, Culbertson CJ, Wolman DN, Mlynash M, Lansberg MG (2021) Hypoperfusion Intensity Ratio Predicts Malignant Edema and Functional Outcome in Large-Vessel Occlusive Stroke with Poor Revascularization. Neurocrit 35(1):79–86. 10.1007/s12028-020-01152-6 10.1007/s12028-020-01152-6 33200332

[20] Kurmann CC, Mujanovic A, Piechowiak EI, Dobrocky T, Zibold F, Beyeler M et al (2022) Heterogeneity of the Relative Benefits of TICI 2c/3 over TICI 2b50/2b67: Are there Patients who are less Likely to Benefit? Clin Neuroradiol 32(3):817–827. 10.1007/s00062-021-01131-0 34989817 10.1007/s00062-021-01131-0 PMC9424153

[21] Alawieh A, Vargas J, Fargen KM, Langley EF, Starke RM, De Leacy R et al (2019) Impact of Procedure Time on Outcomes of Thrombectomy for Stroke. J Am Coll Cardiol 73(8):879–890. 10.1016/j.jacc.2018.11.052 30819354 10.1016/j.jacc.2018.11.052

[22] García-Tornel Á, Requena M, Rubiera M, Muchada M, Pagola J, Rodriguez-Luna D et al (2019) When to Stop. Stroke 50(7):1781–1788. 10.1161/STROKEAHA.119.025088 31177974 10.1161/STROKEAHA.119.025088

[23] Dargazanli C, Consoli A, Barral M, Labreuche J, Redjem H, Ciccio G et al (2017) Impact of Modified TICI 3 versus Modified TICI 2b Reperfusion Score to Predict Good Outcome following Endovascular Therapy. AJNR Am J Neuroradiol 38(1):90–96. 10.3174/ajnr.A4968 27811134 10.3174/ajnr.A4968 PMC7963649

[24] Rodrigues GM, Mohammaden MH, Haussen DC, Bouslama M, Ravindran K, Pisani L et al (2021) Ghost infarct core following endovascular reperfusion: A risk for computed tomography perfusion misguided selection in stroke. Int J Stroke 17(8):897–905. 10.1177/17474930211056228 10.1177/17474930211056228 34796765

[25] Winkelmeier L, Faizy TD, Brekenfeld C, Heitkamp C, Broocks G, Bechstein M, et al. (2023) Comparison of Thrombolysis In Cerebral Infarction (TICI) 2b and TICI 3 reperfusion in endovascular therapy for large ischemic anterior circulation strokes. J Neurointerv Surg10.1136/jnis-2023-020724 10.1136/jnis-2023-020724 PMC11503081 37777256

